# Reinhardt's Adjuster for Fractures of the Lower Jaw

**Published:** 1851-01

**Authors:** 


*Reinhardt's Adjuster for Fractures of the Lower Jaw.*—We have just been shown the above instrument, which, from its facilities of adaptation, and power over this unapproachable portion of the frame, give us ample scope to congratulate the profession upon so valuable and skillful an accession. It consists in a base, shaped like, and grooved for the reception of, the jaw as far back as the angle of the ramus. Through a staple fastened in the center, and projecting beyond the chin, is a steel rod, bent at sufficient length as to pass from below the chin to above the lower teeth, which sustains a plate, fitted to rest upon the teeth, and the lower end for a screw working against the base, so that the plate, when forced, presses them down to a proper position. This plate is held by a screw, for the purpose of using one either for the right or left side, or for the front only. Upon each side is a piece of the same wood, so shaped as to accurately fit the ramus and two-thirds of the jaw, graduated by a slide and screw, working in an upright, which has the advantage of moving, so that the power can be given in any direction, and thus securing any part of the jaw in any position whatsoever, making one of the most complete instruments used for the reduction of any fracture.

Although fractures of the lower jaw are not of very common occurrence, yet, as they do occasionally happen, we take pleasure in calling the attention of the profession to this most ingeniously contrived, and very valuable instrument. It, together with almost every variety of dental and surgical instruments, may be found at Mr. Reinhardt's, Light-st., Balto.

